# Comparative analysis of minimally invasive approaches for gallbladder and common bile duct stones: combined endoscopic techniques vs. ERCP with laparoscopic cholecystectomy

**DOI:** 10.3389/fsurg.2025.1543205

**Published:** 2025-04-30

**Authors:** Haixing Fang, Wenchao Chen, Zhengrong Wu, Guoping Ding

**Affiliations:** Department of General Surgery, Sir Run-Run Shaw Hospital, Zhejiang University, Hangzhou, Zhejiang, China

**Keywords:** choledochoscopy, clinical effect, common bile duct stones, gallbladder stones, gastroscopy, laparoscopy

## Abstract

**Objectives:**

The combined technology of laparoscopy, choledochoscope and gastroscope was used in the treatment of gallbladder stones combined with common bile duct stones, which consists of laparoscopic cholecystectomy (LC), laparoscopic common bile duct exploration with primary closure (LCBDE-PC) and combined gastroscopic and choledochoscopic transabdominal nasobiliary drainage (GC-NBD). The clinical effects of the combined technology were evaluated based on hospital stay, hospital costs, postoperative complications, recurrence of stones, and overall patient satisfaction.

**Methods:**

From July 2017 to December 2020, 206 patients with gallbladder stones combined with common bile duct stones were reviewed retrospectively. According to the surgical method, the patients were divided into Triple-Scope group (LC + LCBDE-PC + GC-NBD), (*n* = 38), ERCP + LC group [endoscopic retrograde cholangiopancreatography (ERCP) followed by LC], (*n* = 96) and T tube group (LC + LCBDE + T tube drainage), (*n* = 72). The differences in stone size, hospital stay, hospital cost, postoperative gallstone recurrence rate and postoperative complications were compared among three groups.

**Results:**

No postoperative bile leak occurred in Triple-Scope group, and patients were discharged successfully, and the abdominal drain was removed around 3 days after surgery, and the nasobiliary drainage was removed around 5 days after surgery with a hospital stay of 9.5 ± 2.65 days. The length of hospital stay and hospital cost in the Triple-Scope group were lower than those in the ERCP + LC group (*P* < 0.01), but not significantly different from those in the T tube group (*P* > 0.05). The diameter of common bile duct and stone size were significantly larger in the Triple-Scope group and T-tube group than in the ERCP + LC group (*P* = 0.001; *P* = 0.004), and the recurrence rate of stones in the Triple-Scope group was not significantly different compared with those in the other two groups (*P* = 0.43).

**Conclusions:**

For patients with gallbladder stones combined with common bile duct stones, the triple-scope combination is safe and effective with fast recovery, and it is worthy of clinical promotion and application.

## Introduction

Gallstones are a common biliary tract disease with an incidence of about 10%, of which 3%–16% may present with concomitant common bile duct (CBD) stones (1–3). In patients with gallbladder stones combined with common bile duct stones, the main surgical options are laparoscopic cholecystectomy combined with laparoscopic common bile duct exploration (LC + LCBDE) or preoperative endoscopic retrograde cholangiopancreatography for removal of common bile duct stones followed by laparoscopic cholecystectomy (ERCP + LC) (4), with both having their own edge (5). It has been shown that with the development of laparoscopic techniques, LC + LCBDE has a smaller stone recurrence rate and less treatment cost than ERCP + LC for patients with gallbladder stones combined with common bile duct stones (1). However, the risk of bile leakage after LCBDE with primary closure (LCBDE-PC) can be as high as 10% even when performed by a skilled surgeon (6). In order to avoid the occurrence of bile leakage, a T-tube is often placed in the common bile duct for drainage, and the T-tube usually needs to be placed for more than 1 month (7, 8), which has a greater impact on the patients' quality of life. Therefore, the search for an innovative minimally invasive procedure based on the LC + LCBDE procedure to reduce or even avoid the occurrence of bile leaks and to make the placement of T-tubes unnecessary for patients has become a common goal pursued by surgeons and patients.

To address the risk of bile leakage after LC + LCBDE-PC, we used an intraoperative protocol of intraoperative nasobiliary drainage placement with a gastroscope combined with a choledochoscope (GC-NBD), which effectively reduced the risk of bile leakage after LC + LCBDE-PC. In this study, we retrospectively analyzed the medical records of 206 patients with gallbladder stones combined with bile duct stones from our hospital between July 2017 and December 2020. We summarized the experience of triple-scope combination (LC + LCBDE-PC + GC-NBD) for gallbladder stones combined with bile duct stones, and compared the outcomes of patients treated with triple-scope combination with those of patients treated with ERCP + LC surgical protocol and LC + LCBDE + T-tube drainage surgical protocol, respectively.

## Materials and methods

### Study population

We retrospectively analyzed the medical records of patients admitted to Sir Run-Run Shaw Hospital, Zhejiang University from July 2017 to December 2020. Patients were included if they met the following criteria. (1) Age >18 years with a confirmed diagnosis of gallbladder stones combined with common bile duct stones based on preoperative ultrasound or MRCP. (2) Common bile duct diameter ≥10 mm as measured by imaging. (3) No history of malignant tumors of the biliary tract or pancreas. (4) No contraindications to laparoscopy, such as severe cardiopulmonary disease or extensive abdominal adhesions.

Exclusion criteria were as follows: (1) MRCP or ultrasound report containing intrahepatic bile duct stones; (2) The preoperative common bile duct diameter was diagnosed by imaging data to be less than 10 mm; (3) History of malignant tumors of the biliary tract and pancreas; (4) Pregnancy.

Patients were divided into ERCP + LC group, T-tube group and Triple-Scope group according to the surgical procedure. In the ERCP + LC group, ERCP was performed first to remove common bile duct stones, followed by LC during the same hospital admission. The two procedures were typically performed within 24–48 h of each other, depending on the patient's condition and surgical schedule. Patients in the T-tube group underwent LC combined with LCBDE and T tube drainage surgical protocol. Patients in the Triple-Scope group underwent LC, LCBDE-PC and GC-NBD surgical protocol.

### Patient data collection

Clinical data were collected from patients including gender, age, imaging data [diameter of common bile duct (cm), maximum diameter of stones (cm), number of stones], preoperative test results (total bilirubin, direct bilirubin, *γ*-glutamyl transpeptidase, and leukocyte count), surgical procedure, length of stay (days), hospital costs, postoperative complications, and patient satisfaction.

### Triple-scope surgical protocol (LC + LCBDE-PC + GC-NBD)

The patient was placed in the supine position after a preoperative fast of 6–8 h. After general anesthesia, the position was adjusted to a head-high to foot-low position of approximately 15°, with the whole body rotated from right to left by approximately 10°, and a sterile towel was laid down. A pneumoperitoneum with a pressure of 12 mmHg was established by making an incision of approximately 10 mm at the infraumbilical margin.

After removal of the gallbladder, a 10-mm longitudinal incision was made in the lower part of the common bile duct, through which a fiberoptic choledochoscope was placed to remove the stones from the common bile duct through a lithotripter basket, or in the case of larger stones, by crushing them with a grasper. After stone extraction, the choledochoscope was reinserted to confirm the patency of the common bile duct and to check that no stones were remained in common bile ducts. A zebra wire was inserted into the common bile duct through an incision below the glabella and through the duodenal papilla to the duodenum. The gastroscope was inserted as usual, and the guide wire was grasped with biopsy forceps and dragged out of the body through the patients' mouth under the gastroscope, through which the nasal bile duct was pushed into the common bile duct. Notably, the nasobiliary drainage tip should be placed more than 1 cm above the common bile duct incision to prevent postoperative bile leakage. The guidewire was removed and the nasobiliary duct was secured beside the patients' ear after confirming its patency by drawing it out of the patients' nasal cavity. The common bile duct was later closed with a full-layer interrupted suture of 4–0 Ethicon absorbable thread with a stitch spacing of 0.3–0.5 cm and a margin of 0.2 cm. Then saline can be injected through the nasobiliary duct or nasocholedochography can be performed to confirm whether the biliary incision was tight. After confirming that the repaired common bile duct was tightly closed, an abdominal drainage tube was placed near the repaired common bile duct and the abdomen was routinely closed.

The nasobiliary tube used in the Triple-Scope procedure had an external diameter of 7 Fr and a length of 150 cm. The tube was placed with its tip positioned at least 1 cm above the common bile duct incision to ensure effective drainage and reduce the risk of bile leakage.

After primary closure of the common bile duct, a saline solution was gently infused through the nasobiliary tube under low pressure. The absence of fluid extravasation at the suture site confirmed the integrity of the closure. If leakage was detected, additional sutures were placed to ensure a watertight closure. This method provided a simple and effective way to verify the adequacy of the repair before completing the surgery.

### ERCP + LC protocol

In the ERCP + LC group, patients first underwent ERCP for the removal of common bile duct stones. ERCP was performed under sedation, and a sphincterotomy was typically performed to facilitate stone extraction using a basket or balloon catheter. After successful stone removal, patients underwent LC during the same hospital admission, usually within 24–48 h. This two-stage approach avoids the need for T-tube placement but may be associated with complications such as pancreatitis, cholangitis, or sphincter of Oddi dysfunction (9).

### T-tube protocol

In the T-tube group, patients underwent LC combined with LCBDE. After stone extraction, a T-tube was placed in the common bile duct for external drainage. The T-tube was typically left in place for 4–6 weeks postoperatively to ensure adequate biliary decompression and to allow for postoperative cholangiography to confirm the absence of residual stones. Although effective, T-tube drainage is associated with patient discomfort, electrolyte imbalances, and the risk of retrograde infections (10).

### Definitions of complications and recurrent stone

#### Biliary leakage

Defined as the presence of bilirubin-rich fluid in the abdominal drain or imaging evidence of fluid collection with confirmed bile content. Severity is classified as follows. Mild: Asymptomatic, managed conservatively. Moderate: Requires endoscopic or percutaneous intervention. Severe: Necessitates surgical re-intervention or leads to life-threatening complications.

#### Pancreatitis

Defined as inflammation of the pancreas, diagnosed based on clinical symptoms (e.g., abdominal pain), elevated serum amylase or lipase levels (≥3 times the upper limit of normal), and imaging findings. Severity is graded according to the revised Atlanta classification.

#### Cholangitis

Defined as an infection of the biliary tract, typically presenting with Charcot's triad (fever, jaundice, and right upper quadrant pain) or Reynolds' pentad (Charcot's triad plus hypotension and altered mental status). Severity is classified based on the Tokyo Guidelines.

#### Wound infection

Defined as the presence of purulent discharge, erythema, warmth, or tenderness at the surgical site, with or without positive microbiological culture. Severity is categorized as superficial, deep, or organ/space infection.

#### Recurrent stone

Defined as the reappearance of biliary stones in the gallbladder, bile ducts, or intrahepatic ducts after a confirmed complete clearance, as evidenced by imaging studies (e.g., ultrasound, CT, or MRCP).

### Follow-up

All patients were followed up at 1 year postoperatively through telephone interviews, outpatient reviews, and imaging studies (e.g., ultrasound or MRCP), and all patients were followed up until September 2021. The immediate follow-up period (within 30 days postoperatively) focused on detecting early complications such as bile leakage, wound infection, or pancreatitis. Late follow-up (up to 1 year) aimed to identify stone recurrence or biliary stricture. Bile leakage: Detected by abdominal drain output analysis or imaging studies (e.g., CT or ultrasound). Stone recurrence: Confirmed by imaging studies showing new stones in the biliary tract. Other complications: Diagnosed based on clinical symptoms, laboratory tests, and imaging findings.

### Statistical analysis

All data were analyzed by SPSS Version 18.0. Age and gender data, preoperative biochemical indexes, common bile duct diameter, number of stones, stone size, length of hospital stay, hospital costs, postoperative complication rate, and patient satisfaction were counted for both groups. The measurement data were described as mean ± standard deviation, and the differences among groups were analyzed by one-way ANOVA. The between-group differences of the count data were tested by chi-square test. Differences among groups were considered statistically significant if *P* < 0.05.

## Results

### Pre-operative patient information

According to the treatment modality, patients who met the enrollment criteria were divided into 3 groups, including 96 patients in the ERCP + LC group, 72 patients in the T-tube group, and 38 patients in the Triple-Scope group, and the baseline characteristics of the included patients were shown in Table 1. There was no significant difference in gender between the Triple-Scope group and the ERCP + LC group (*P* = 0.547), and the age was younger than that of the ERCP + LC group (*P* < 0.01). The diameter of common bile duct and stone size were significantly larger in the Triple-Scope group and T-tube group than in the ERCP + LC group (*P* = 0.001; *P* = 0.004), and there was no significant difference in the number of stones compared with the ERCP + LC group (*P* = 0.1). There were no significant differences in total bilirubin, direct bilirubin, *γ*-glutamyl transpeptidase and leukocyte count in the Triple-Scope group compared with patients in the ERCP + LC group (All *P* > 0.05).

**Table 1:** Baseline characteristics of the included patients.

Variables	ERCP + LC group (*n* = 96)	T-tube group (*n* = 72)	Triple-Scope group (*n* = 38)	F	*P*
Gender					0.547
Man	51	30	18		
Woman	45	42	20		
Age	61.7 ± 17.03	58.1 ± 15.41	53.7 ± 9.82	116	<0.01*
Common bile duct diameter (Mean ± SD)	1.14 ± 0.395	1.36 ± 0.461	1.56 ± 0.554	10.92	0.001*
Stone size (Mean ± SD)	0.91 ± 0.589	1.27 ± 0.819	1.34 ± 0.670	8.22	0.004*
Number of stones (Mean ± SD)	2.05 ± 1.33	2.2 ± 0.99	2.4 ± 0.94	2.73	0.1
Total bilirubin (Mean ± SD)	44.0 ± 42.39	32.3 ± 33.04	46.4 ± 35.87	3.865	0.051
Direct bilirubin (Mean ± SD)	25.8 ± 29.49	16.6 ± 25.68	23.8 ± 26.32	3.807	0.053
*γ*-Glutamyl transpeptidase (Mean ± SD)	377.1 ± 349.40	320.9 ± 373.94	382.1 ± 318.42	1.84	0.176
Leukocyte count (Mean ± SD)	7.1 ± 7.74	6.3 ± 3.44	6.21 ± 4.32	3.17	0.076

* *P* < 0.05.

### Treatment results

All patients in the Triple-Scope group successfully completed the expected operation without any reversion to open surgery and no change of T-tube drainage, and the operation time was 130–210 min, with an average of 162.8 ± 78.32 min; the bleeding volume was 5–50 ml, with an average of 38.5 ± 30.62 ml; all patients were discharged without any bile leakage. All patients had their abdominal drains removed 3–5 days after surgery, and their nasobiliary ducts were removed 5–8 days postoperatively. Before removal of the nasobiliary ducts, nasobiliary angiography was performed to confirm that no stones remained and that the contrast agent entered the duodenum successfully.

The treatment results of the three groups were shown in Table 2. The length of stay and hospital costs were significantly less in the Triple-Scope group than in the ERCP + LC group (9.5 ± 2.65 days *vs.* 12.1 ± 6.53 days, *P* < 0.01; 32,634 ± 10,327 RMB *vs.* 34,842 ± 11,823 RMB, *P* < 0.01), and there was no significant difference compared with the T-tube group; the recurrence rate of stones in the Triple-Scope group was not significantly different compared with those in the other two groups (*P* = 0.43).

**Table 2:** Comparison of treatment results.

Variables	ERCP + LC group (*n* = 96)	T-tube group (*n* = 72)	Triple-Scope group (*n* = 38)	F	*P*
Hospitalization time (Day)	12.1 ± 6.53	10.8 ± 6.60	9.5 ± 2.65	12.8	<0.01*
Hospitalization fees (¥)	34,842 ± 11,823	31,881 ± 18,441	32,634 ± 10,327	11,638	<0.01*
Recurrence of stones (Cases)	9	4	2		0.43

* *P* < 0.05.

### Postoperative complications

The postoperative complications in the three groups were shown in Table 3. There was no postoperative death in any of the three groups. 2 cases (2.08%) of postoperative pancreatitis occurred in the ERCP + LC group, and the symptoms resolved after 2 weeks of treatment with conservative therapy such as octreotide. In the T-tube group, 2 cases (2.78%) of wound infection and 1 case (1.39%) of biliary duct infection were observed, which were resolved after 1–2 weeks of regular dressing changes and corresponding antibiotic treatment. One case (2.63%) of wound infection occurred in the Triple-Scope group, and the wound was healed after 1 week after regular dressing changes were given.

**Table 3:** Postoperative complications and management of the included patients.

Variables	ERCP + LC group (*n* = 96)	T-tube group (*n* = 72)	Triple-Scope group (*n* = 38)	Management
Bile Leakage	0	0	0	Fasting
Pancreatitis	2 (2.08%)	0	0	Conservative treatment such as growth hormone inhibitors
Wound infection	0	2 (2.78%)	1 (2.63%)	Dressing changes
Cholangitis	0	1 (1.39%)	0	Antibiotic Treatment

### Satisfaction survey

We conducted a satisfaction survey during the postoperative follow-up. Very satisfied and basic satisfaction were defined as total satisfaction. As shown in Table 4, the total satisfaction in the Triple-Scope group and the ERCP + LC group was similar, with individual patients giving unsatisfactory ratings due to the occurrence of postoperative complications, but the T-tube group had the lowest total satisfaction, with three of the unsatisfied patients having reasons related to complications, and the remaining three patients were dissatisfied because postoperative discharge with a T-tube caused some inconvenience to their lives, and it was hoped that improvements could be made.

**Table 4:** Satisfaction survey of the included patients.

Variables	ERCP + LC group (*n* = 96)	T-tube group (*n* = 72)	Triple-Scope group (*n* = 38)
Very satisfied	70	35	30
Basic satisfaction	22	31	7
Total satisfaction	92 (95.84%)	66 (91.7%)	37 (97.37%)
Dissatisfaction	4 (4.16%)	6 (8.3%)	1 (2.63%)
Reasons for dissatisfaction	Postoperative complications (4)	Discharged with T-tube (6) Postoperative complications (3)	Postoperative complications (1)

## Discussion

In 1941, Smyth (11) first reported the use of common bile duct exploration for stone extraction and T-tube drainage for the treatment of common bile duct stones. With the advent of laparoscopic techniques, LCBDE has become the mainstream for the treatment of intra- and extrahepatic common bile duct stones. In 1991, LCBDE was first reported in patients with common bile duct stones (12).With the widespread use of LCBDE, postoperative complications due to unskilled operation have gradually decreased (13). Cai et al. (1) showed that compared to ERCP + LC procedure for gallbladder stones combined with common bile duct stones, LC + LCBDE had better treatment results with lower stone recurrence rate, lower hospital cost, and lower length of stay. However, after surgical treatment of LC + LCBDE, patients often need to be discharged with a T-tube, which not only seriously affects their quality of life, but also predisposes them to complications such as water-electrolyte disturbances, massive bile loss, and retrograde infections (10).

ERCP is widely used in the treatment of common bile duct stones (14–17), and the stones in the common bile duct can be removed in advance after preoperative ERCP extraction, and only LC removal of the gallbladder is required for the second-stage surgery. ERCP + LC can avoid the inconvenience of postoperative T-tube placement and the discomfort of skin pruritus caused by T-tube. However, loss of sphincter function after ERCP + LC is often caused by disruption of the integrity of the Oddi's sphincter, which can lead to duodenal reflux, and postoperative complications such as cholangitis or even pancreatitis may also occur (9, 18–20). Moreover, the stones removal rate of ECRP often depends on the proficiency of the endoscopists, and requires the endoscopists' extensive experience in dealing with a variety of complex biliary physiological variants, which often results in complications such as stone remnants for the inexperienced endoscopist.

In contrast to ERCP + LC, the Triple-Scope procedure, which requires a higher level of experience in ERCP operation, uses a choledochoscope that allows stone visualization and other conditions for stone extraction. Meanwhile, the placement of a nasobiliary tube under the gastroscope is simple and convenient compared to the complexity of ERCP. The nasobiliary ducts can be used not only for intraoperative leak detection of the common bile duct suture opening, but also for postoperative drainage and pressure reduction to avoid bile leakage caused by excessive pressure in the common bile duct.

Our findings align with previous studies demonstrating the superiority of LCBDE over ERCP + LC in terms of stone recurrence rates, hospital costs, and length of stay (21). However, the triple-scope technique further improves upon traditional LCBDE by eliminating the need for T-tube drainage, thereby enhancing patient comfort and quality of life. The shorter hospital stay and lower costs observed in the Triple-Scope group compared to the ERCP + LC group underscore the economic and clinical benefits of this approach.

Notably, the triple-scope technique appears to be particularly suitable for patients with larger common bile duct stones or more complex biliary anatomy, as evidenced by the significantly larger stone size and bile duct diameter in the Triple-Scope and T-tube groups. This suggests that the technique may fill an important niche in the management of challenging cases where ERCP may be less effective.

In order to avoid the inconvenience of T-tube drainage, some surgeons have tried LC + LCBDE-PC procedure, but the high incidence of postoperative bile leakage is inevitable. Liu et al. (18) found that the overall incidence of bile leak was 11.3% in 141 patients with common bile duct stones treated with LC + LCBDE-PC procedure, with a significantly higher incidence of 31.6% in patients with common bile duct diameters <1 cm. This showed that the major disadvantage of the LC + LCBDE-PC procedure was the high incidence of bile leakage, which was one of the key factors affecting the success of this procedure.

Compared with LC + LCBDE-PC, the placement of nasobiliary duct in the Triple-Scope procedure not only eliminates the inconvenience of carrying a T-tube after the first-stage bile duct suture, but also improves the safety of the first-stage bile duct suture by avoiding the bile leakage caused by excessive pressure in the bile duct after the suture, which brings a more comfortable and reliable treatment experience for patients. Compared to single-stage laparoscopic cholecystectomy with intraoperative ERCP (LC + IO-ERCP), the Triple-Scope technique offers distinct advantages in managing complex CBD stones. While LC + IO-ERCP avoids nasobiliary drainage, its efficacy heavily depends on endoscopists' expertise and real-time coordination between surgical and endoscopic teams (22). The Triple-Scope approach should be considered due to its direct visualization of stones via choledochoscopy, which is particularly advantageous for large or multiple stones (23).

In this study, we also counted the data of postoperative complications in the three groups of patients. All three groups had individual complications postoperatively, including pancreatitis, wound infection, and cholangitis, with the least postoperative complications (one wound infection) in the Triple-Scope group, which may be related to the smaller number of patients enrolled in the Triple-Scope group. The present study had some limitations with a small sample number, and a large sample is needed for confirmation in the future.

Despite its promising results, this study has several limitations that warrant consideration. First, the retrospective design introduces the potential for selection bias, and the relatively small sample size of the Triple-Scope group limits the generalizability of the findings. Second, the follow-up period may not be sufficient to fully assess long-term outcomes, such as stone recurrence or biliary stricture. Future prospective, randomized controlled trials with larger sample sizes and extended follow-up periods are needed to validate these findings and further refine the indications for the triple-scope technique.

Additionally, future research could explore the integration of advanced imaging modalities, such as intraoperative cholangiography or real-time ultrasound, to enhance the precision of stone clearance and reduce the risk of residual stones. The development of standardized protocols for nasobiliary tube placement and postoperative management could further optimize outcomes and facilitate the broader adoption of this technique.

In conclusion, this retrospective study showed that triple-scope procedure has the advantage of shorter hospital stay and reduced hospital costs compared with ERCP + LC. The triple-scope procedure provided a higher quality of life and treatment experience for patients compared with LC + LCBDE + T-tube drainage procedure. For patients with gallbladder stones combined with common bile duct stones, the triple-scope procedure has the advantages of convenience, safety and comfort, and is a recommended procedure in clinical practice.

## Data Availability

The datasets presented in this study can be found in online repositories. The names of the repository/repositories and accession number(s) can be found below: The data can be uploaded upon reasonable request to corresponding author.
